# Cationised Fibre-Based Cellulose Multi-Layer Membranes for Sterile and High-Flow Bacteria Retention and Inactivation

**DOI:** 10.3390/membranes13030284

**Published:** 2023-02-27

**Authors:** Vanja Kokol, Monika Kos, Vera Vivod, Nina Gunde-Cimerman

**Affiliations:** 1 Faculty of Mechanical Engineering, Institute of Engineering Materials and Design, University of Maribor, Smetanova 17, 2000 Maribor, Slovenia; 2 Department of Biology, Biotechnical Faculty, University of Ljubljana, Jamnikarjeva 101, 1000 Ljubljana, Slovenia

**Keywords:** fibrous membrane, cationised cellulose nanofibrils, amino-hydrophobised cellulose nanofibrils, antibacterial activity, multi-layer structure, flux, bacteria retention

## Abstract

Low-cost, readily available, or even disposable membranes in water purification or downstream biopharma processes are becoming attractive alternatives to expensive polymeric columns or filters. In this article, the potential of microfiltration membranes prepared from differently orientated viscose fibre slivers, infused with ultrafine quaternised (qCNF) and amino-hydrophobised (aCNF) cellulose nanofibrils, were investigated for capturing and deactivating the bacteria from water during vacuum filtration. The morphology and capturing mechanism of the single- and multi-layer structured membranes were evaluated using microscopic imaging and colloidal particles. They were assessed for antibacterial efficacy and the retention of selected bacterial species (*Escherichia coli*, *Staphylococcus aureus*, *Micrococcus luteus*), differing in the cell envelope structure, hydrodynamic biovolume (shape and size) and their clustering. The aCNF increased biocidal efficacy significantly when compared to qCNF-integrated membrane, although the latter retained bacteria equally effectively by a thicker multi-layer structured membrane. The retention of bacterial cells occurred through electrostatic and hydrophobic interactions, as well as via interfibrous pore diffusion, depending on their physicochemical properties. For all bacterial strains, the highest retention (up to 100% or log 6 reduction) at >50 L/h∗bar∗m^2^ flow rate was achieved with a 4-layer gradient-structured membrane containing different aCNF content, thereby matching the performance of industrial polymeric filters used for removing bacteria.

## 1. Introduction

Microfiltration is a pressure- or vacuum-driven separation technique that uses a micrometre-sized porous membrane as a barrier. Bacterial cells (with sizes ranging from 0.1 to 10 µm) are removed from the fluid by physical size-exclusion rejection, and, optionally, electrostatic interception (repulsion and/or adsorption) mechanisms. This occurs either through surface screening or depth filtration, depending on the physicochemical properties of the membrane, such as surface charge, hydrophobicity, porosity and the characteristics (morphology, surface charge, type of cell envelope) of the bacterial cells [1]. Such filtration processes are important steps in wastewater pre-treatment and portable water purification, as well as in initial biopharmaceutical downstream processes (before expensive filtration and chromatographic separation systems) and in the food industry (for clarification of fermented beverages) [2,3]. Therefore, disposable low-cost porous filter membranes, appropriate for use in the initial stages of purification processes due to their ability to retain microbial cells (with at least log 6 of reduction, or 99.9999% percentage reduction), have received considerable attention recently.

Nevertheless, the non-ionic (synthetic or semi-synthetic) polymer filters, such as PES (polyethersulphone), PA (polyamide), PVDF (polyvinylidene difluoride), PP (polypropylene), CA (cellulose acetate) and cellulose nitrate (CN), are still primarily used [2]. The main reasons for this are their high retention, non-/low-swelling, and low biomolecule/proteins-adsorbing properties, although they might be affected by buffer, operating conditions and sterilisation processes. Additionally, the chemical selectivity of surface-modified polymeric membranes can be affected by different solute–surface interactions (e.g., hydrophobic, ionic, hydrogen bonding, etc.) and they are neither biodegradable, renewable, or cost-effective.

In the past, significant efforts have been made to develop colloidal filtration (mainly sand, glass bead, etc.) based on the physicochemical interactions between colloids and media. The major limitation was the diminished retention capacity of colloidal particles within the pore spaces and low filtration rate [4]. Recently, low-cost polymeric alternatives have been investigated with increasing frequency. These have been composed from microporous electrospun nanofibre nonwovens of various microporous structures, thickness and surface chemistry. These are produced from biobased (such as, e.g., chitosan/PEO, chitosan/PEO/TEMPO-oxidised cellulose; [5,6]) up to synthetic (such as, e.g., polyacrylonitrile/PAN) polymers [7]. However, in order to increase their efficacy in removing bacteria, these alternatives have to be stabilised and surface-modified. Another novel approach is represented by thin-film and multi-layered nanocomposite membranes. In this case, the first barrier interfacial polymerised layer (such as, e.g., antibacterial active PAN/chitosan) is complemented by a polymeric electrospun mat acting as the second layer (for removal of bacteria and other micro-pollutants) and a polymeric nonwoven layer as the third layer (to provide mechanical strength) [8]. Such a composited membrane displayed up to 99.9999% retention of bacteria at minimum fouling, but enabled only low water flux (with up to a few minutes of contact time).

Textile materials have also emerged as low-cost filter substrates for the removal of colloidal particles [9], due to 10-fold higher filtration velocities as compared to granular media [10]. The attachment of bacteria on such a nonwoven fabric depends primarily on the media-suspension particles’ interaction, being the most effective by the hydrophobic surfaces. Their efficiency also depends on the bacterial cell concentration, fluid velocity (influenced by thickness, areal density and pore size of the random fibrous web), as well as the presence of divalent salts and water pH. Most reports on the filtration of bacteria with textile materials are based on the use of a fabric as a substrate. In the only available study using textile fibres, the authors showed the importance of PA fibres’ orientation on such a filter performance due to the change in the contact and collision (attachment) efficiency of the fibres [11].

Nanocellulose has been also recognised as an emerging renewable, sustainable and cost-efficient material for the removal of microbes [1,12,13,14,15,16]. Cellulose nanopapers, produced from cellulose nanofibrils (CNF) by traditional paper-making processes, have been studied in various membrane filtration applications [17], both in size-exclusion, as well as in ion-exchange/adsorption operations, including for the removal of viruses [14,18,19,20], thereby matching the performance of industrial synthetic polymer filters. A CNF-based membrane filter was surface-modified with polyelectrolyte multi-layers using cationic polyvinyl amine and anionic polyacrylic acid [21]. However, the performance of such filters is limited, due to their low permeability caused by nano-scale porosity and physical weakening (swelling) in the presence of water. These problems can be overcome by enhancing the cellulose hydrophobicity, increasing mechanical strength, or by cross-linking using, e.g., citric acid [22]. The ultra-fine and surface-modified (i.e., carboxylated or cationised) CNF was also integrated into an electrospun polymeric microfiltration membrane [23] or shaped into highly porous foam by cryo-templating [24], given its high (i.e., log 6) bacterial cell retention capability, while exerting antibacterial activity against Gram-negative *Escherichia coli*, respectively. Anyway, limited studies were reported for the removal of other types of bacterial species from water using nanocellulose-based filtration material.

In this study we investigated the efficacy of simple fibre-based filter membranes, functionalised with integrated and quaternised and amino-hydrophobised CNFs for the removal of bacteria from an aqueous suspension using vacuum filtration. In our previous study [25], we showed that CNF bearing molecules with amino group and hydrophobic moiety increase electrostatic and hydrophobic interactions with non-polar bacterial cells, resulting in outstanding bacteriostatic and bactericidal activity (up to 100% or 10 log reduction at ≥8 mg/L) against both Gram-positive (G+) *Staphylococcus aureus* and Gram-negative (G−) *Escherichia coli*. The focus of this study was, therefore, on the potential improvement of the bacteria adsorption and filtration of single- and multi-layer structured filter membranes by adding cationised CNF, and, at the same time, also evaluate their structure (density, porosity) using microscopic imaging and colloidal particles. In addition, during vacuum-assisted filtration, the retention ability was assessed for three bacterial species: *E. coli* (G−), *S. aureus* (G+) and *Micrococcus luteus* (G+), differing in cell envelope structure, hydrodynamic biovolume and arrangement of the cells. 

## 2. Materials and Methods

### 2.1. Materials and Chemicals

The 100% viscose (VIS) fibres of 1.3 dtex and 39 mm long were prepared as compact fibre slivers of 5.1 kTex fineness by Litija Spinnery Ltd. Slovenia), according to the conventional industrial process (mixing and carding), and used for the membrane preparation.

The quaternised cellulose nanofibrils (qCNF) of 2–3 μm long and 10–200 nm wide highly branched fibrils with around 0.23 degree of substitution, as determined by conductometric titration, were prepared by Xylocel Oy, from Finland, according to patent WO/2016/075370 [26] using glycidyltrimethylammonium chloride. The wood-based chain-like CNFs, with diameters in the 10–70 nm range and lengths of few micrometres (1–3 µm), were purchased from the University of Maine, The Process Development Center in the USA, and were used to prepare amino-hydrophobised CNF (aCNF) using hexamethylenediamine (HMDA). The hexamethylenediamine (HMDA, 98% purity) and all other chemicals used were purchased from Sigma-Aldrich Co., Ltd. (Merck, Slovenia). All chemicals were of an analytical grade and used without additional purification. 

Spherical and monodispersed polystyrene (PS) particles of different dimeters (0.5 ± <0.05 µm and 2.0 ± <0.1 µm) and surface chemistry (plain (95585) and carboxylic surface-modified and core-labelled with red fluorophores (L3030 and L3280) were purchased from Sigma-Aldrich. The plain particles were negatively charged colloidal particles, stabilised with terminal sulphate groups on their surface. These were received as 2% solid content and dispersed in 0.1–0.5% surfactant and 0.2% NaHCO_3_ and K_2_SO_4_. The surface-modified and labelled particles were received as 2.5% solid content, labelled with 0.2–0.6% dye and contained 0.1% NaN_3_ as a preservative. 

Cultures of the Gram-negative (G−) bacterial strain *Escherichia coli* (EXB-V127) and the Gram-positive (G+) bacterial strains *Staphylococcus aureus* (EXB-V54) and *Micrococcus luteus* (EXB-V52) were obtained from the Culture Collection Ex of the Infrastructural Centre Mycosmo, Department of Biology, Biotechnical Faculty, University of Ljubljana, Slovenia (https://www.ex-genebank.com/index.php/en/ (accessed on 20. 12. 2022)). The bacterial strains were nonpathogenic, nonsporulating, chemoorganotrophic and aerobic. All tests with bacteria were performed under aseptic conditions with sterile materials and chemicals.

### 2.2. Preparation of Amino-Hydrophobised CNF (aCNF)

The functionalisation of the CNF was performed by a two-step procedure, where the hydroxyl groups at the C2 and C3 in the glucose rings of cellulose chains were first oxidised to aldehyde functional groups by periodate oxidation, and in the second step they were functionalised/conjugated with HMDA by the Schiff base reaction, as described in our previous study [25,27]. Briefly, aqueous dispersions of CNF (1 wt%, 100 mL) and pre-dissolved sodium periodate (1.6 g NaIO_4_/g CNF) were mixed and stirred for 48 h in the absence of light (protected with aluminium foil) at room temperature. The products were washed thoroughly several times with deionised water, before being functionalised with HMDA. The functionalisation of the pre-oxidised CNF suspension (200 mL of 0.5 wt%) was performed with the addition of 8 mmol of HMDA. The mixture was stirred continuously for 6 h at 30 °C. This was followed by the in situ reduction of the resulting imine intermediate at room temperature, employing 0.58 g of NaBH_4_. After stirring for 3 h, the product was washed thoroughly several times with deionised water until a neutral pH was reached and was then used as prepared. The total charge of aCNF, determined by potentiometric titration, was around 4.57 mmol/g at pH 7. 

### 2.3. Assessment of Hydrodynamic Size and Zeta Potential of the qCNF, aCNF, and PS Particles

The hydrodynamic size and zeta potential of the qCNF, aCNF and differently modified PS particles, suspended in milli-Q water at a concentration of 0.01 wt%, were assessed by Dynamic Light Scattering equipment (DLS, Zetasizer, Nano ZS ZEN360, Malvern Instruments Ltd., Malvern, UK) at 25 ± 0.1 °C. The researchers used the DTS1070 disposable folded capillary cell, applying the following parameters: a material refractive index of 1.47 (cellulose) or 1.59 (PS particles), a dispersion refractive index of 1.33 (milli-Q water), and viscosity of 0.8872 cP (milli-Q water). A field of 150 V was applied across the nominal electrode spacing of 16 mm. The samples (1 mL) were measured immediately after being dispersed them at 10,000 rpm for 3 min using an Ultra-turrax T25 (IKA GmbH, Staufen, Germany). The average values were calculated from at least four individual measurements.

### 2.4. Preparation of Fibre Membranes

Individual (i.e., mono-layer) membranes were prepared from 4 viscous (VIS) slivers laid alternately perpendicular and diagonally in different directions, and cut into disks of approximately 47 mm in diameter. Each of the membrane layers (of approximately 0.43 g) was impregnated with (a total of) 50 mL of 0.1 wt% water-suspended qCNF (i.e., 50 mg or 1 mg/mL), with or without the addition of 0.1–0.5 wt% aCNF (i.e., 50–250 mg or 1–5 mg/mL), and left immersed for 24 h. The qCNF/aCNF suspensions were dispersed with either a magnetic stirrer or the Ultra-turrax T25 homogenizer (IKA GmbH, Staufen, Germany) at 10,000 rpm for 3 min before use. In addition, a special set of membranes were prepared from four VIS slivers. Each was impregnated with a differently concentrated aCNF suspension, and, finally, combined into a single (mono-layer) composite membrane, referred to as a mixture/MIX. The membranes were then dewatered by vacuum-assisted filtration using a cellulose filter paper (MN 651, Macherey-Nagel GmbH and Co. KG, Dueren, Germany) with an active filtration radius of 110 mm as a support, and then cold-pressed between two aluminium foils at 5 bar and room-temperature conditions for 24 h. Before pressing, another viscous sliver was added onto the surface of the membrane to prevent sticking to the aluminium foil and, thus, losing any of the CNF. The fibre membranes were stored in the dark at 4 °C until microbiological testing had been performed. 

The membranes were tested in two ways, namely, as individual (mono-layer) membranes, or as multi-layer “sandwich” membranes. This was carried out by stringing 2–4 mono-layer membranes of the same or different types (each impregnated with differently concentrated aCNF suspension) on top of each other. For a better understanding of all the membranes tested, a summary of the samples and their mode of testing is listed in Table 1.

### 2.5. Characterisation of the Fibre Membranes

#### 2.5.1. Membrane Mass, Thickness, and Density

All the membranes were conditioned for 24 h at standard conditions according to ISO 139:2005 [28] (temperature of 20 ± 2°C and relative humidity of 65 ± 5%) before the testing. The membrane mass was measured, and the mass per unit area (g/m^2^) was calculated. The mass changes (Δm) were also assessed by using Equation (1):

Δm = (m_before_ − m_after_)/m_before_ × 100 (%) (1)

where m_before_ and m_after_ are the mass of the membrane sample before and after the filtration, respectively. The thickness of the membrane samples was evaluated according to the standard ISO 5084:1996 [29], using a thickness gauge for textiles and rubber (Hildebrand, Germany), and the density (ρ) was calculated from the weight and thickness values by using Equation (2):

(2) $$\rho =\frac{m}{t}\;(g/{\mathrm{cm}}^{3})$$

where m is the mass (g/m^2^) and t is the fabric thickness (cm). At least two measurements were performed, and the average value was reported for each sample.

#### 2.5.2. Membranes’ Morphology

The membrane morphology (structure, homogeneity/distribution) before and after filtration of bacterial suspensions was determined using scanning electron microscopy (SEM) imaging and optical microscopy. The SEM imaging of the samples’ surfaces and cross sections was performed using a FEI Sirion 400NC (FEI, Hillsboro, Oregon, USA) microscope at different magnifications. The membranes tested in the filtration experiments were immersed in 30 mL of 70% ethanol twice consecutively for half an hour in order inactivate viable bacterial cells and to fix them. Then, they were transferred to sterile Petri dishes and left to dry for 3 days at 37 ± 0.5 °C before further preparation for SEM analysis. 

For light microscopy, selected non-tested membranes were cut into smaller square pieces (approx. 5 mm × 5 mm) and immersed in deionised water for 15 min at room temperature. Afterwards, the membrane pieces were dissected into individual slivers, which were transferred onto an objective glass, and observed under an Olympus SZ61TR stereomicroscope equipped with an Olympus DP73 camera (Olympus, Tokyo, Japan). 

### 2.6. Membranes’ Filtration Performance Using Micro-Spherical Polystyrene (PS) Particles

The milli-Q water suspended (0.1 mg/L) spherical and monodispersed PS particles of different dimeters (ø = 0.5 and 2 µm) and surface chemistry (plain, negatively/carboxylated surface-modified and core-labelled with red fluorophore) were filtered (25 mL) through the membranes at 0.6 bar pressure by the dead-end filtration mode using a Sterlitech HP4750 stirred cell (Auburn, Washington, DC, USA) and 2.24 cm^2^ of effective surface area. The permeate solutions were collected, and their UV-Vis (at 410 nm as pre-determined from the scan in the range of 190–800 nm) or fluorescence (at an excitation of 540 nm and emission of 542 nm) intensity were measured before and after filtration using a spectrophotometer (Tecan Infinite M200, Männedorf, Switzerland) at PS particles’ relevant wavelengths. The water and solution fluxes, as well as the removal percentage efficacy, were also determined using Equation (3) and Equation (4), respectively:

Flux rate = V/(A × t × p)     (in L/h∗bar∗m^2^) (3)

Removal = (C_before_ − C_after_)_before_ × 100    (in %) (4)

where V is the filtrate volume (L), t is the time of individual filtration (h), p is the vacuum of the filtration (bar), A is the area of the membrane filter (m^2^), while C_i_ and C_t_ are the particles’ concentrations (g/L) before and after filtration, respectively. 

A sample (permeate) collected after each filtration was also analysed by DLS using a Zetasizer in order to quantify the difference in concentration between the feed and the permeate and calculate the rejection vs. adsorption rate of the PS particles. In addition, the fluorescence intensity was evaluated on the top (feeding side) and the bottom sides of each membrane after the filtration of fluorophore-labelled PS particles by multiple (100 ×) and well-aligned (5 × 5) readings in an area size of 0.4 µm using a spectrophotometer. Results were expressed in relative fluorescence units (RFU). The filtration experiments were repeated at least twice at each set of conditions, with a fresh membrane used for each experiment.

### 2.7. Membranes’ Filtration Performance Using Bacteria Cell Suspensions

#### 2.7.1. Preparation of Bacterial Cell Suspensions

For all experiments, the bacterial strains were revitalised and maintained on standard tryptic–soy–agar (TSA, tryptone 15 g/L, soy peptone 5 g, NaCl 5 g/L, agar 15 g/L, pH 7.2) anfd incubated at 37 ± 0.5 °C for 24 h (*E. coli*) to 48 h (*S. aureus* and *M. luteus*). Individual colonies were then inoculated into 50 mL of tryptic–soy–broth (TSB) and incubated overnight (for 16 h *E. coli* and *S. aureus*; and for 18 h *M. luteus*) at 37 ± 0.5 °C on a rotary shaker (Innova 230, New Brunswick Scientific, Enfield, CT, USA) at 180 rpm. The ice-cooled overnight bacterial biomass was harvested by centrifugation at 6000 rpm and 4 ± 0.5 °C for 5 min. The cell pellet was rinsed twice by resuspension in deionised water, followed by another centrifugation. The final pellet was then resuspended in deionised water and the optical density was measured at 600 nm (UV Spectrometer 1800, Shimadzu, Kyoto, Japan). The bacterial cell concentration was adjusted to ~10^8^ CFU/mL. For the microfiltration experiments, a calibrated suspension of bacterial cells was diluted 100 times in the required volume of deionised water. The final concentration of bacterial cells in the feed suspension was ~10^6^ CFU/mL. Its homogeneity throughout the experiment was maintained using a magnetic stirrer. 

#### 2.7.2. Filtration Performance Assay with Bacterial Cell Suspensions

The microfiltration performance of the fibre membranes was assessed by the vacuum filtration method using a vacuum/pressure pump (VCP80, VWR, Leuven, Belgium) and a vacuum filtration system (MilliporeSigma, Merck KGaA, Darmstadt, Germany). The filtration system consisted of a 1000 mL borosilicate Erlenmeyer flask for the collection of the permeate and a glass filter holder unit, comprising 300 mL borosilicate glass funnel, a sintered membrane filter holder (for 47–50 mm filters) with a silicone stopper and an aluminium spring clamp. Prior to microbiological testing, the membranes were autoclaved for 15 min at 121 ± 0.5 °C (Systec DX65, Systec, Linden, Germany). An individual sterile membrane was inserted into the assembled sterile filter system and preconditioned by filtering 300 mL of deionised water at 0.2–0.6 bar and room temperature in order to provide an even wetness and constant water flux. Immediately after wetting, 200 mL of the bacterial suspension was filtered through individual or multi-layered sandwich membranes at 0.6 bar and room temperature. Up to 7 (on average 4) consecutive filtrations of the feed suspension were performed for each membrane type in order to evaluate their performance in terms of permeability and microfiltration efficiency for the removal of bacteria.

The concentration of bacteria in the feed suspension and filtrate after each filtration was determined by the plate count method. All the samples were subjected to 10-fold serial dilutions (10^−1^–10^−6^) in physiological saline, plating in duplicates on the TSA, and incubation at 37 ± 0.5 °C for 24 h (*E. coli*) or 48 h (*S. aureus* and *M. luteus*). After incubation, the number of colonies on countable plates was counted (30–300 colony forming units, CFU), and the average CFU/mL was calculated. The filtration efficiency for the removal of bacteria from the feed suspension was expressed as a log reduction factor (Equation (5)) and reduction percentage (Equation (6)). These parameters were calculated using the following equations, where N_0_ and N are the feed and filtrate bacteria concentrations in the CFU/mL, respectively:

(5) $$\mathrm{Log}\;\mathrm{reduction}\;\mathrm{factor}=\mathrm{Log}\;\left(\frac{N_{0}}{N}\right)$$

(6) $$\mathrm{Reduction}\;\mathrm{percentage}=\frac{{(N}_{0}-N)}{N_{0}}\times 100\;(\mathrm{in}\;\%)$$

In addition, the water permeation and bacterial suspension flux rates were calculated using Equation (3).

### 2.8. Assessment of Antibacterial Properties

#### 2.8.1. Assessment of the Antibacterial Properties of qCNF and aCNF Suspensions

The antibacterial properties of the qCNF and aCNF suspensions were tested with *E. coli* (EXB-V127) and *S. aureus* (EXB-V54) via the broth macrodilution antibiogram. Bacterial cultures were prepared as described previously in 2.7.1. An overnight preculture (4 mL) was inoculated into 50 mL of fresh TSB and incubated for 3 h at 37 ± 0.5 °C on a rotary shaker (Innova 230, New Brunswick Scientific, Enfield, CT, USA) at 180 rpm. The ice-cooled bacterial biomass was harvested and used to prepare the inoculum, which was added directly to the test tubes containing 3 mL of TSB (control) or serially diluted qCNF or aCNF suspensions in TSB. The initial concentration of bacterial cells in the test tubes was ~10^6^ CFU/mL. First, two-fold dilutions of the autoclaved and homogenised qCNF and aCNF suspension stocks (1.2 wt% or 12 mg/mL qCNF; 3.2 wt% or 32 mg/mL aCNF) were prepared in double-strength TSB, and then diluted further in single-strength TSB to obtain 2-fold to 16-/64-fold serial dilutions, respectively (for detailed information, see Supplementary Table S1). After 24 h incubation at 37 ± 0.5 °C on a rotary shaker at 180 rpm, the tubes were inspected visually for turbidity (indicating bacterial growth) and viability by the plate count method. All the samples were subjected to 10-fold serial dilutions (10^−1^–10^−9^) in physiological saline, plated in duplicates on the TSA, and incubated at 37 ± 0.5 °C for 24 (*E. coli*) to 48 h (*S. aureus*). After incubation, the number of colonies was counted (30–300 CFU) and the average CFU/mL was calculated. The endpoints for evaluating the antibacterial effect of the qCNF and aCNF suspensions were survival (bacterial cell viability) and growth (cell proliferation). Test suspensions were tested in two independent experiments, and the results were reported as average (±Standard Deviation, SD) values of the log reduction (Equation (5)) and the percentage reduction (Equation (6)), minimum inhibitory concentration (MIC), and minimum bactericidal concentration (MBC). The MIC defines the lowest concentration of the antibacterial agent that inhibits bacterial growth, while MBC defines the lowest concentration of the antibacterial agent that results in microbial death [30]. If the exact MIC could not be determined, because broth discoloration and increased turbidity from the dispersed samples masked or resembled bacterial growth [30], the MIC was reported as an MIC range between the first non-inhibitory concentration and the MBC. 

#### 2.8.2. Assessment of Antibacterial Properties of the Selected Fibre Membranes

The antibacterial properties of selected individual mono-layer membranes, non-impregnated (control) and those impregnated with 0.1% qCNF, both with and without the addition of 0.3% aCNF, were tested on *E. coli* (EXB-V127) by the absorption method and a bacterial viability assay. 

The absorption method was carried out according to the international standard for the determination of the antibacterial activity of textile products (ISO 20743:2013, [31]). Briefly, an individual colony was inoculated into 20 mL of TSB and incubated for 18 h at 37 ± 0.5 °C on a rotary shaker at 180 rpm. An overnight preculture (0.4 mL) was inoculated into 20 mL of fresh TSB and incubated for 3 h under the same conditions. The ice-cooled bacterial biomass was harvested by centrifugation at 6000 rpm and 4 ± 0.5 °C for 5 min. The cell pellets were rinsed twice with deionised water. A test inoculum was prepared in 20-times diluted TSB, and the cell concentration was adjusted to 1.0–3.0 × 10^5^ CFU/mL. An individual membrane disc was cut into equal quarters, weighed (~0.2 g), placed in separate vials, and autoclaved. Then, 0.2 mL of the inoculum was applied at several points on each of the specimens. Immediately after the inoculation, 20 mL of the neutralising solution (Tween 80 30 g/L, lecithin 3 g/L, histidine hydrochloride 1 g/L, meat peptone 1 g/L, NaCl 4.3 g/L, KH_2_PO_4_ 3.6 g/L, Na_2_HPO_4_.2H_2_O 7.2 g/L) was added into two control and two test vials, and mixed thoroughly with vortex mixture. The remaining vials were capped loosely and incubated for 24 h at 37 ± 0.5 °C. After incubation, 20 mL of the neutralising solution was added into the vials and mixed thoroughly by vortexing. The bacterial concentration in the shake-out suspensions was determined by the plate count method. All the samples were subjected to 10-fold serial dilutions (10^−1^–10^−9^) in physiological saline, plated in duplicates on the TSA, and incubated at 37 ± 0.5 °C for 24 h. After incubation, the number of colonies on countable plates was counted (30–300 CFU), and the average CFU/mL, log reduction factor (Equation (5)), and reduction percentage (Equation (6)) were calculated. In addition, the antibacterial activity value (A) (Equation (7)) was calculated as the difference between the growth value on the control specimen (F) and the growth value on the membrane specimen (G):

A = F − G = (log C_t_ − log C_0_) − (log T_t_ − log T_0_) (7)

where log C_t_ and log C_0_ are the common logarithms of the arithmetic average of the bacteria count obtained from the non-impregnated membrane specimens after 24 h incubation and immediately after inoculation (time 0 h), respectively; and log T_t_ and log T_0_ are the common logarithms of the arithmetic average of the bacteria count, obtained from the impregnated membrane specimens after 24 h incubation and immediately after inoculation (time 0 h), respectively.

The bacterial viability and bactericidal potential of the membranes against *E. coli* (EXB-V127) was tested further with the LIVE/DEAD BacLight Bacterial Viability Kit (L7012, Invitrogen, Molecular probes), as described previously in [32], with some modifications. Briefly, after filtration of 200 mL of the feed suspension (~10^6^ CFU/mL) through an individual membrane, the feed suspension and filtrates were stained with a mixture of two fluorescent dyes (1:1 *v*/*v*), the SYTO9 (3.34 mM) and the propidium iodide (PI, 20 mM). The dye mixture was added directly to the feed suspension and filtrate; the final concentrations of the SYTO9 and PI in the staining solution were 5 µM and 30 µM, respectively. The membranes were first cut into smaller squares (8 × 8 mm), placed individually into the wells of 24-well flat-bottom test plates (TPP, Trasadingen, Schwitzerland), and covered with 1 mL of the dye mixture, which was prepared in deionised water. The stained suspensions and membranes were incubated at room temperature in the dark for 30 min. After incubation, blank solutions (deionised water and the dye mixture in deionised water) and unstained and stained bacterial cell suspensions were pipetted (1 mL) into separate wells of 24-well flat-bottom test plates. The unstained and stained membrane pieces were first washed twice with deionised water, transferred into the wells and covered with 1 mL of fresh deionised water. All the samples were tested in triplicates, and the data are shown as the average value (±SD). The fluorescence was measured spectroscopically with a Biotek Cytation Hybrid Multimode Reader (Agilent, Santa Clara, CA, USA) using an excitation wavelength centred at 475 nm, while the relative fluorescence intensities (RFU) were acquired of the green (G; Syto9 emission at 505 nm) and red (R; PI emission at 617 nm) emissions. Live bacterial cells (i.e., cells with intact cell membranes) were stained green, whereas dead cells or cells with damaged membranes were stained red. In parallel with the fluorescent staining, the concentration of bacteria in the feed suspension and filtrate after filtration was determined by the plate count method. 

## 3. Results and Discussion

### 3.1. Affinity of Bacteria to a qCNF and aCNF Infused Fibre Membrane

**Figure 1:**
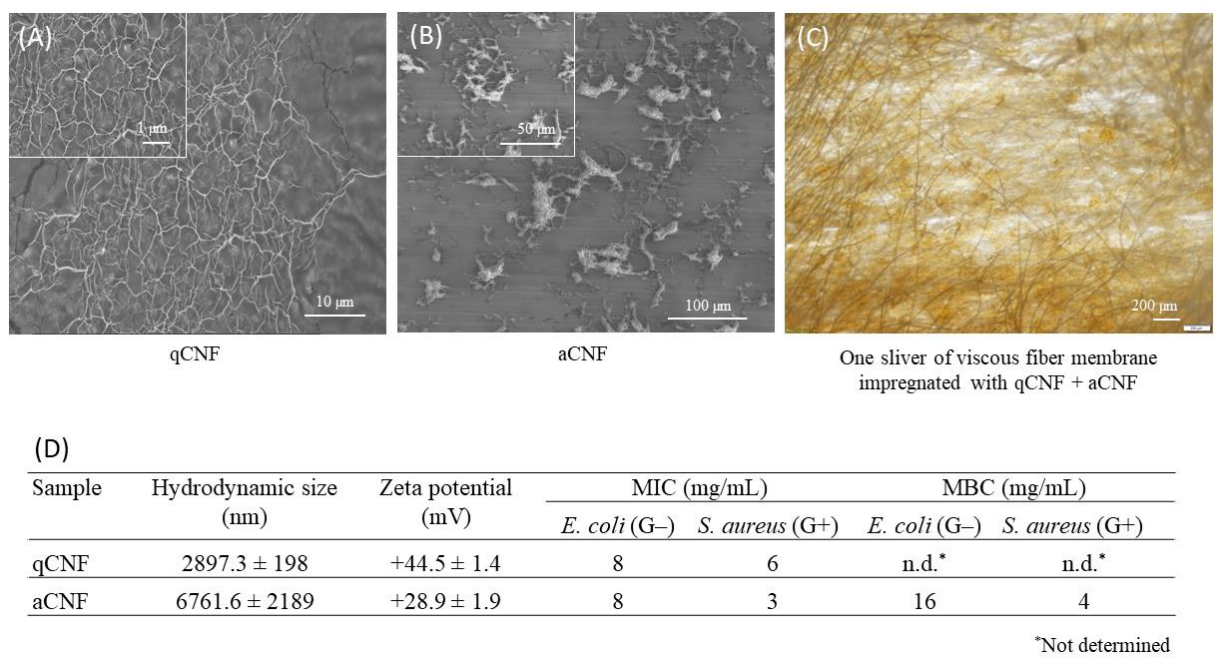
Selected data on the characterisation of the quaternised cellulose nanofibrils (qCNF) and amino-hydrophobised cellulose nanofibrils (aCNF) dispersed (0.01 wt%) in milli-Q water (pH 6.7): (**A**,**B**) SEM micrographics, (**D**) hydrodynamic size, and (**D**) zeta potential. (**D**) Minimum inhibitory (MIC) and minimum bactericidal (MBC) concentrations of the qCNF and aCNF suspensions for Gram-negative (G−) bacterium *Escherichia coli* (EXB-V127) and Gram-positive (G+) bacterium *Staphylococcus aureus* (EXB-V54) were determined by the broth macrodilution antibiogram method. (**C**) The optical image presents the dense matrix of viscous fibres and CNF in one sliver, that was isolated from the one-layer membrane impregnated with 0.1 wt% qCNF and 0.3 wt% aCNF.

The difference in the morphological structure of water-suspended qCNFs vs. aCNFs can be distinguished clearly from the SEM images presented in Figure 1, showing thin and highly polydispersed qCNF fibrils and porous micro-sized (partly aggregated) aCNFs. As can be seen from Table embedded in Figure 1, both qCNF and aCNF suspensions exhibited antibacterial potential in the 24-h broth macrodilution test, the use of which resulted in major, concentration-dependent reductions in survival (viability) and growth (reproduction) against *E. coli* (EXB-V127) and *S. aureus* (EXB-V54). The average MIC values of qCNF (8 mg/mL for *E. coli* and 6 mg/mL for *S. aureus*, respectively) were similar or lower than the MIC values of the aCNF (around 8 mg/mL for *E. coli* and around 3 mg/mL for *S. aureus*). Antibacterial agents are usually regarded as bactericidal if the MBC is not more than four times the MIC [30]. In our study, the aCNF suspensions could be considered both bacteriostatic and bactericidal since the MBC values were not more than twice as high as the MIC. 

The observed differences between qCNFs and aCNFs might be related to both the type (i.e., pH-dependent dissociated amino groups vs. ionised *quaternary* ammonium groups), the quantity and accessibility of cationised groups, as well as their zeta potential and hydrophobic character; the latter was more pronounced for aCNF due to the presence of hexamethyl chains. Both types of CNFs can, thus, possibly play an essential role in the interaction with the outermost layer of the bacterial cell envelope [33,34]. The cell envelope is a complex and dynamic multi-layered structure which confers the shape and rigidity of the cell, plays a variety of protective and adaptive roles against physical, chemical, and biological factors, and participates in the adhesion processes [35,36,37]. Structurally and biochemically, it differs markedly in (G−) and (G+) bacteria, which can influence their interaction and susceptibility to various agents. This was demonstrated in the case of the (G+) strain *S. aureus* (EXB-V54), which was more susceptible to both tested materials as compared to the (G−) strain *E. coli* (EXB-V127). Briefly, a (G−) cell envelope is composed of three layers: the inner cytoplasmic membrane, the thin peptidoglycan cell wall (only a few nanometres thick) and the outer membrane. The envelope is composed of phospholipids and lipoproteins in its inner leaflet and lipopolysaccharides in its outer leaflet [35,37]. On the other hand, the (G+) cell envelope comprises two layers, the inner cytoplasmic membrane and adjacent thick (30–100 nm) peptidoglycan cell wall [35,36,38], outwardly enriched with accessory versatile anionic glycopolymers (such as teichoic acids, teichuronic acids, and other acidic or neutral polysaccharides) [39]. 

However, irrespective of the cell wall type, the presence of acidic and basic functional groups, associated with lipopolysaccharides and phospholipids, in the outer membrane of (G−) bacteria or peptidoglycans, along with the presence of accessory glycopolymers in the cell wall of (G+) bacteria, contribute to an overall negatively charged bacterial cell surface [35,36]. For illustration, the average zeta potentials, an electrochemical property of the cell surface of untreated bacterial cells in 0.5 mM potassium phosphate-buffered solution (pH 7.4), were −44.2 mV for *E. coli* (MTCC 2939) and −35.6 mV for *S. aureus* (MTCC 96) [40]. Charged functional groups at bacterial cell surfaces influence their electrostatic behaviour in response to the conditions in the immediate environment, and, thus, regulate the cells’ adhesion processes and interactions with various substrates [40,41]. The hypothesis is that the positively charged functional groups of qCNF and aCNF can associate with the negatively charged bacterial envelope, resulting in disruption of the cell wall, defects of the cytoplasmic membrane permeability, and, consequently, severe leakage of intracellular low molecular weight compounds [8,42]. In addition, the adhesion of hydrophilic bacteria (having a low static contact angle of water, namely, around 30° for *E. coli* O157:H7 (ATCC 700728) and −33° for *S. aureus* (ATCC 13368) generally increases with more hydrophobic abiotic surfaces [41], which may restrict transport to the inner cytoplasmic membrane [33,34]. The diminished MIC/MBC effect of qCNF as compared to aCNF can thus be explained by a general lack of interactions between the bacterial cells and the available quarternised groups of hydrodynamically smaller (on average around 2.89 vs. 6.76 μm, respectively) and less hydrophobic qCNFs. On the other hand, the relatively better interactions with antibacterially active aCNFs can be interpreted by a stronger and diverse (electrostatic, hydrophobic and unspecific) adsorption of more hydrophobic and protonated aCNF to the negative surface-charged and unprotonated bacterial surfaces [25,33,34]. In addition, the relatively longer hydrophobic chains of HMDA seem to be more compatible with the bacterial cell wall, possibly facilitating diffusion and ultimately leading to the disruption of the cytoplasmic membrane and cell death [25,33,34].

**Figure 2:**
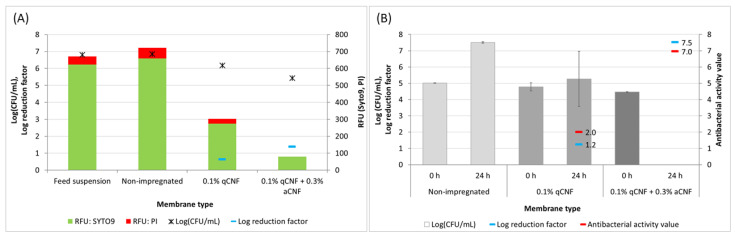
Viability of Gram-negative *Escherichia coli* (EXB-V127) after (**A**) acute exposure during the filtration experiment, and (**B**) chronic exposure to selected individual membranes, namely, non-impregnated membrane (control) and membranes impregnated with 50 mL of 0.1% quaternised cellulose nanofibrils (qCNF) with or without the addition of 0.3% amino-hydrophobised CNF (aCNF). (**A**) The viability after acute exposure was determined by the SYTO9 and propidium iodide (PI) fluorescence viability staining with a LIVE/DEAD BacLight Bacterial Viability Kit (L7012, Invitrogen, Molecular probes) and the plate count method. The fluorescence was measured spectroscopically by using an excitation wavelength centred at 475 nm. The stacked bar graph shows the average relative fluorescence units (RFU) of the green (Syto9, at 505 nm) and red (PI, at 617 nm) emissions after subtracting the signal of the dye solution without cells (blank) from the stained bacterial cell suspensions. (**B**) The chronic 24-h contact exposure to selected individual membranes was determined by the absorption method (ISO 20743:2013, [31]). The results are presented as the average values of log (CFU/mL) (±SD) and log reduction factor (primary *x* axis). In addition, the antibacterial activity value, calculated as the difference between the growth value on the non-impregnated fibrous membrane and the qCNF/aCNF impregnated membrane specimens, is also given (secondary *x* axis).

However, the concentrations of the qCNF/aCNF suspensions (i.e., 1/1–5 mg/mL or 50/50–250 mg/membrane) used for the impregnation of the individual fibrous mono-layer membranes were lower (*E. coli*) or similar (*S. aureus*) to the concentrations (8 and 3 mg/mL, respectively) that affected the growth (reproduction) and viability of bacterial cells when exposed continuously under the test conditions of the 24-h macrodilution antibiogram. In order to evaluate the impact of qCNF/aCNF on the antibacterial properties of the membranes, selected mono-layer membranes, namely, non-impregnated and impregnated with 0.1% qCNF (1 mg/mL, 50 mg) with or without the addition of 0.3% aCNF (3 mg/mL, 150 mg), were tested in conditions of acute (bacterial viability assay) and chronic (absorption assay, ISO 20743:2013, [31]) contact exposure to *E. coli* (EXB-V127). The results, presented in Figure 2A (and additionally supported also by Supplementary Figure S1), showed that the acute short-term exposure of *E. coli* (EXB-V127) during filtration through individual membranes had no significant effect on their viability, both in terms of the cells that passed through the membranes into the filtrate suspension and the cells that were captured successfully by the membranes. The ratio between the relative fluorescence intensities (RFU) of the green (Syto9 emission at 505 nm; live cells) and red (PI, emission at 617 nm; damaged or dead cells) emissions of the stained bacteria in the filtrates showed that the great majority of the bacteria in the population are represented by viable cells, whereas a significantly smaller signal was found to have come from the damaged and/or dead cells. This was expected, since the bacterial biomass was prepared from a 16-h stationary-phase culture characterised by equilibrium between the numbers of dividing and dying cells [43]. The RFU values also corresponded well to the number of viable cells (CFU) determined by the plate count method. The non-impregnated fibre membrane had no retention ability, while both impregnated membranes had retention potential, successfully reducing the number of bacterial cells in the filtrates The log reduction factor thus increased by about 2.2 times, i.e., ranging from 0.63 for the membrane impregnated with 0.1% qCNF alone, to 1.38 for the membrane also containing 0.3% aCNF. 

On the other hand, the chronic 24-h contact exposure of *E. coli* (EXB-V127) to the selected membranes (Figure 2B), determined by the absorption method (ISO 20743:2013, [31]), showed distinct and material-specific responses. The non-impregnated membrane did not exhibit any antibacterial activity, while the membrane impregnated with 0.1 wt% qCNF showed strong bacteriostatic potential (i.e., the bacteria survived, but did not reproduce) with minor bactericidal effects (log reduction 1.2 and 94.3% percentage reduction). In the case of the membrane impregnated with both 0.1% qCNF and 0.3% aCNF, a strong bactericidal effect was confirmed, which was shown as a complete reduction of the exposed bacterial cells (i.e., log reduction 7.5 and 100% percentage reduction). The antibacterial activity values for both treated VIS membranes, 0.1 wt% qCNF with or without 0.3% aCNF, were 2.0 and 7.0, respectively, and these can be considered as materials with strong to significant antibacterial properties (ISO 20743:2013, [31]), respectively.

### 3.2. Filtration Performance of Spherical PS Microparticles Using Single-Layer Membranes with Different aCNF Content

The surface biochemical characteristics of the bacterial cells (charge and hydrophobicity of the cell envelope) differed significantly, and, thus, influenced their surface-to-volume ratio (hydrodynamic biovolume, influenced by the size, shape and arrangement of the cells), as well as interactions with the membrane matrices [35,36,37]. The filtration performance in terms of permeability and retention of bacteria, is, thus, in addition to the pore size distribution and porosity (or density) of the membrane, also affected directly by the surface affinity (adsorption) principle and fibrous membrane swelling. 

The round-shaped (G+) *S*. *aureus* bacteria are about three times smaller (0.5–1 μm in diameter) than the rod-shaped (G−) *E*. *coli* (~0.5–1 μm in diameter and 2–5 μm in length) [44,45]. In order to mimic the differently arranged round-shaped bacteria and to verify the size-exclusion and ion adsorption filtration principle of the differently prepared membranes, dead-end filtration was performed (at 0.5 bar) of spherical and overall negatively surface-charged PS particles of different sizes or hydrodynamic volumes (plain of 0.5 µm/−68.7 mV; carboxylated of 0.5 µm/−17.5 mV, and 2.0 µm/−33.7 mV). In addition, the water permeation was assessed, and the membranes’ morphological structures were evaluated by high-resolution microscopic imaging and corresponding areal and volumetric densities. 

**Figure 3:**
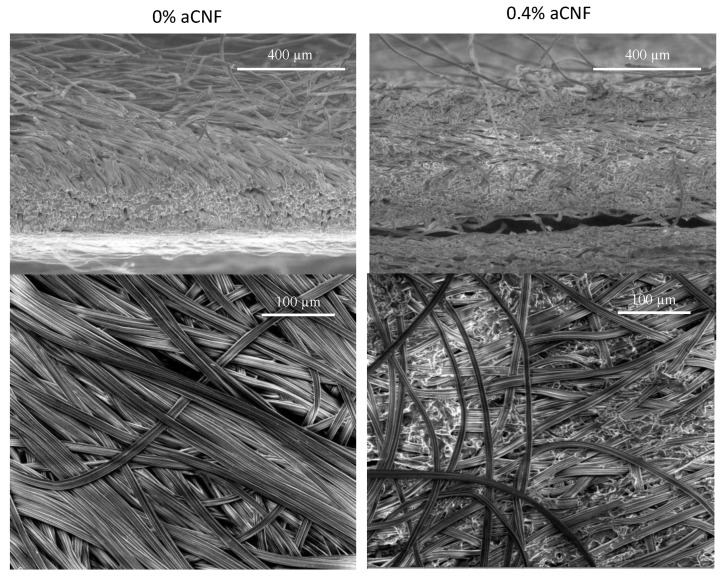
SEM micrographs of the cross section (**top**) and surface (**bottom**) of differently prepared individual (single-layered) fibrous membranes, namely, impregnated with 0.1 wt% quaternised cellulose nanofibrils (qCNF), with or without the addition of 0.4 wt% amino-hydrophobised CNF (aCNF).

**Figure 4:**
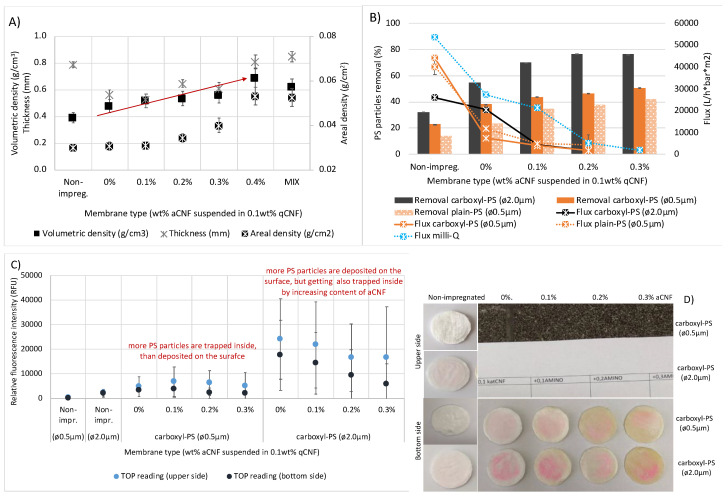
Dependence of the PS particle hydrodynamic diameter and zeta potential (surface-carboxylated PS of 0.5 µm/−17.5 mV and 2.0 µm/−33.7 mV, and plain-PS of 0.5 µm/−68.7 mV) on (**A**) physical characteristics and (**B**) dead-end filtration performance (removal and flux rate) of differently prepared (impregnated with different wt% of amino-hydrophobised CNF/aCNF, pre-suspended in 0.1 wt% quaternised cellulose nanofibrils/qCNF) individual (single-layered) membranes, performed by the dead-end filtration mode at 0.6 bar. (**C**) The relative fluorescence multiple-reading intensities (RFU) on the membranes’ upper and bottom sides after the filtration of different fluorescence-labelled carboxyl-PS particles, with (**D**) photographs of the tested membranes after filtration of the carboxyl-PS particles.

The microstructure of different densities is clearly visible in the surface-related and cross-sectional morphology (Figure 3) of the individual mono-layer fibrous membranes when prepared with or without 0.4 wt% aCNF and predispersed in 0.1 wt% qCNF. The distribution of aCNF through the fibrous membrane was reflected in smaller open structures (interfibrillar/fibre voids) with less densely packed and oriented viscose fibres. Their areal density (Figure 4A) increased from 0.03 to 0.05 g/cm^2^ and the thickness from 0.56 to 0.81 mm, resulting in the increase in volumetric densities from 0.47 to 0.68 g/cm^3^, which benefited the removal of larger (2 μm) and carboxylated spherical PS particles (Figure 4B). An increasing number of particles was captured by the higher content of aCNF, while the smaller ones (i.e., 0.5 μm, both plain and carboxylated-PS particles) diffused into the smaller pores/voids, slowing the flow through the membrane and the flux rate and confirming the size-exclusion principle of filtration. As the 0.5 μm large carboxyl-PS particles were trapped better than their counterparts (plain PS), the ionic-interaction principles were also included. The deposition of fluorescence-decorated carboxyl-PS particles on the membrane (upper/feed and bottom) surface sides, recorded optically (Figure 4D) and by measuring the Relative Fluorescence Intensity (RFU) on their surfaces after filtration experiments (Figure 4C), showed a generally higher intensity of a red colour and RFU values (i.e., more particles) on the membrane upper surfaces by using larger (2 μm) particles and their reduction on the bottom side with increasing the content of aCNF in the membrane. On the other hand, membranes filtered with the same volume and concentration of smaller (0.5 μm), although quantitatively four times higher in derived count rate values (obtained from DLS by Zetasizer) than larger (2 μm) PS particles, showed a much lighter colour (i.e., less particles) on both sides of the membranes, additionally confirming their entrapment into the membranes’ inner structure. This was also supported by a decrease in the flux rate of PS particles’ suspensions through the aCNF-impregnated membrane, which was more pronounced in the case of the quantitatively higher 0.5 μm large, both plain and carboxyl-PS particles. In comparison, in the case of only qCNF impregnated membranes (i.e., 0% aCNF) the larger (2 μm) PS particles were captured on the surface rather than after penetrating into the interior of the membrane, suggesting that the surface filtration mechanism, which was slightly reduced when the membranes were also impregnated with aCNF, increased the filtration time by more than once. 

### 3.3. Water Permeation Using Individual Membranes with Different aCNF Content

**Figure 5:**
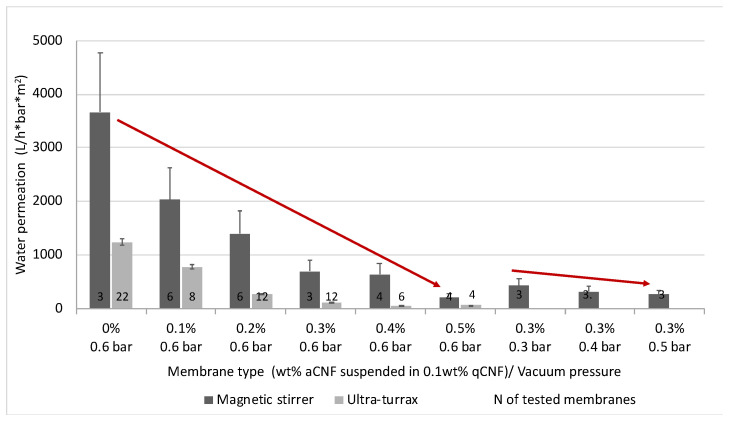
The effect of differently prepared (impregnated with different wt% of amino-hydrophobised CNF/aCNF, pre-suspended in 0.1 wt% quaternised cellulose nanofibrils/qCNF by magnetic stirring or Ultra-turrax) individual (single-layered) membrane on permeation of (300 mL) deionised water by vacuum filtration at different pressures (0.6–0.2 bars). The water permeation of the pristine/unmodified membrane could not be measured due to an excessively fast water permeation rate, and is therefore not included. The numerical marks on the bottom of the bars present the number of tested membranes.

In order to evaluate the effect of the membrane surface affinity (adsorption) and viscose fibres/CNF fibrils’ swelling, the permeation of (300 mL) deionised water was conducted on the selected single-layer membranes by vacuum filtration (as performed in the case of bacteria filtration) at different pressures (0.6–0.2 bar), and the flow rates were determined. The preliminary results showed that the method of dispersing CNF had a great influence on their redistribution into the internal fibre structure of the membrane, and, consequently, on the flow rate (Figure 5). The water permeability decreased in proportion to the content of qCNF and aCNF and their dispersion, as well as due to increased vacuum pressure. The membrane, when impregnated at a filtration pressure of 0.6 bar with (magnetic stirring dispersed 0.2 wt% and 0.3 wt% of aCNF, decreased in permeability by 60.4% (from ~39,138 to ~15,507 L/h∗bar∗m^2^) and, further, 94.5% (to ~214 L/h∗bar∗m^2^) compared to the membrane impregnated only with 0.1 wt% of qCNF (i.e., 0% aCNF). The SEM micrographs (Figure 3) and results of the membrane densities (Figure 4A) showed a reduction of the open pores/fibrous interstices and increase in the overall density after the integration of CNFs, due to the web formation between the viscose fibres and their gluing. At the same time, the intrinsic properties of qCNFs, above all its highly hydrophilic and water retention ability, due to the presence of an abundant quantity of –OH groups, increased the wettability of the membranes significantly, making them more compacted and also hydrophilic, and thus resulting in the reduction of water permeation. This effect was even more pronounced by the addition of aCNF, which also bore highly polar amino functional groups that contributed additionally to the surface hydrophilicity, as already confirmed in our study [46], although with lower water absorption capacity due to the presence of longer hydrophobic moieties from the HMDA molecules which were conjugated to the CNF. It was obvious that the water flow through the fibrous interstices was not straight but was directed by the content and distribution of aCNF blocking it, reducing the available space for its flow. However, the permeation values obtained in this study are still comparable to other similar studies and thus show a potential for application. Previous studies showed that electrospun cellulose acetate impregnated with a small amount (3 wt%) of hydrophilic cellulose nanocrystals attained only at a pressure of 6–8 bars (CNCs) does not have an effect on water permeance, which is also increasing with increasing pressure (from 5000 to 22,000 L/h∗bar∗m^2^ at 0.5–2 bars), while high CNC loading (45 wt%) reduced it to 500–5000 L/h∗bar∗m^2^ [47]. The graphene oxide (GO) deposited on the freestanding CNF membrane surface [48] was also found to reduce CNF swelling and allow more stable and relatively high (8000 L/h∗bar∗m^2^) water permeance, comparable to pure Nylon 66 of similar (0.2 µm) porosity. On the other hand, the wettability of hydrophobic polyethersulfone membranes was increased by coating with CNC and TEMPO-oxidised (carboxylic) CNF, while the water permeability remained unaffected (around 3100 L/h∗bar∗m^2^) [49]. 

### 3.4. Retention Efficacy of Bacteria Using Individual Membranes with Different aCNF Content

**Figure 6:**
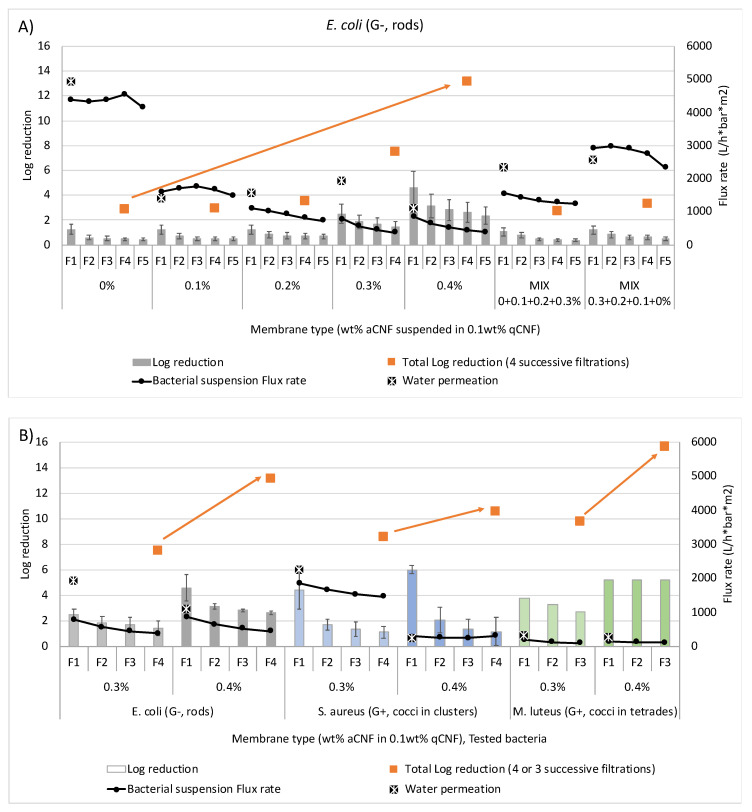
The effect of differently prepared (impregnated with different wt% of amino-hydrophobised CNF/aCNF, pre-suspended in 0.1 wt% quaternised cellulose nanofibrils/qCNF) individual (single-layered) membrane on permeation of (300 mL) deionised water and flux rate of bacterial suspension with log reduction values for (**A**) rod-shaped (G−) *Escherichia coli* (EXB-V127) vs. (**B**) round-shaped (G+) *Staphylococcus aureus* (EXB-V54) and (G+) *Micrococcus luteus* (EXB-V52), performed with vacuum filtration at a pressure of 0.6 bar.

The stationary-phase (G−) rods of relatively hydrodynamically larger *E. coli* (EXB-V127) were used as model bacterial cells to study the effect of aCNF content (0.1–0.4 wt%) in a single-layered fibrous membrane on bacterial removal efficiency. The membranes were initially wet prior to the first filtration of bacterial suspension, and the water permeation was measured. As shown in Figure 6A, increasing the aCNF content from 0.1 to 0.4 wt% decreased the water permeation significantly, which also increased the retention efficiency of *E. coli* after the first filtration cycle (F1) from a log reduction of 1.20 to 4.57, respectively. The retention of *E. coli* was only slightly dependent on the aCNF content until the addition of 0.2 wt% aCNF, while the flux rate was reduced by 35.3% (from about 1700 to 1100 L/h∗bar∗m^2^) but did not change significantly after further increasing the aCNF content to 0.4 wt% (to about 868 L/h∗bar∗m^2^). This suggests that the retention of bacteria during filtration depends on the interfibrillar/fibre voids available for their diffusion into smaller pores during filtration through which water can diffuse. With each subsequent filtration cycle (from F1 to F4), both the log reduction and flux rate decreased gradually, reaching a log reduction of 2.32 (−49.2%) by the forth filtration cycle (F4), although the flux rate (386 L/h∗bar∗m^2^) was lower, due to more retained bacterial cells. This affected the reduction in pore size and available surface area on the aCNF/qCNF matrix for specific surface-based interaction/adsorption or entrapment of bacteria, thus fulfilling different filtration modes [1]. At the beginning of filtration, only larger pores/open spaces between the fibres/fibrils were available, while smaller pores were not involved in the filtration. However, the latter contributed to the filtration performance of the membrane after a longer filtration time. In the case of a single-layer (MIX) membrane, in which the aCNF content increased gradually from sliver to sliver (in total 0.6 wt% of aCNF), the samples did not show an additional increase in the bacterial retention, as expected, but were similar to the samples with up to 0.2 wt% aCNF. This was probably due to the excessively short contact time of bacterial cells with the membrane during filtration. 

Apart from the membrane porosity, bacterial cells may be prevented from passing through the membrane due to electrostatic and (even stronger) hydrophobic interactions with the membrane material, as well as the physicochemical properties of both the membrane and the bacterial cells [2]. As shown in Figure S2, which presents the morphology of Gram-stained stationary-phase bacterial cells of the test strains, the size, shape and arrangement of bacterial cells differed significantly: *E. coli* (EXB-V127) is characterised by single rod-shaped cells with rounded ends (bacilli; 0.5–1 μm in diameter and 2–5 μm in length), whereas the cells of the other two bacteria are spherical (cocci) and tend to be arranged in groups of different sizes. Namely, *S. aureus* (EXB-V54; 0.5–1 μm in diameter) forms mostly irregular grape-like clusters, and *M. luteus* (EXB-V52; 0.5–3.5 μm in diameter) forms tetrads, which can aggregate in larger clusters [44,45,50]. However, as shown in Figure 6B, the removal efficiency of all the tested bacterial strains was always the highest during the first (F1) filtration cycle (i.e., log reduction between 2.5 and 6.0), and then decreased gradually with each subsequent filtration (up to a log reduction of 1.11–2.7 at F4), regardless of the membrane type (i.e., the aCNF content), with the decrease in the corresponding flux rates being almost negligible. The effect of bacterial retention was strongly dependent on species. It was more pronounced for relatively hydrodynamically smaller (0.5–1.0 μm) *S*. *aureus*, for which the log reduction decreased by approximately 74.7% (from 4.4 to 1.11) and even 80.6% (from 6.0 to 1.16) when membranes with higher (0.4 wt%) aCNF content were used, followed by rod-shaped (G−) *E*. *coli*. Relatively high efficiencies independent of filtration cycles (log reduction 3.78–2.70 for 0.3 wt% aCNF and log reduction 5.20 for 0.4 wt% aCNF), but comparatively much lower flux rates (250–108 L/h∗bar∗m^2^), were measured for the spherical, relatively hydrodynamic and largest tetrades of (G+) *M. luteus*. This showed that the performance of the tested membranes in terms of filtration efficiency, as well as flowability, depended significantly on the properties of the surfaces of bacterial cell clusters, i.e., on the homogeneity of the distributed surface charge, and, thus, on the electrostatic attraction or repulsion [2], and on their ability to change physiologically (in shape, size, and/or biovolume). It was also revealed that the performances align when subjected to mechanical stress and osmotic/transmembrane pressure during filtration [51]. Thus, more flexible, spherical cells (such as of *S. aureus* and *M. luteus*) were able to align better with the water flow, and therefore diffuse directly into the smaller interfibrillar/fibre pores [52], in contrast to the rod-shaped cells (such as of *E. coli*) that encounter the filter pores randomly [53]. Moreover, the stressed bacterial cells have smaller cells and are therefore able to pass through the filter open spaces without difficulty [53], which was the case for each subsequent filtration cycle of *E. coli*. On the other hand, this effect was hardly observed for larger tetrades of (G+) *M. luteus* [54], resulting in a good retention, even at the lowest flow rate. 

**Figure 7:**
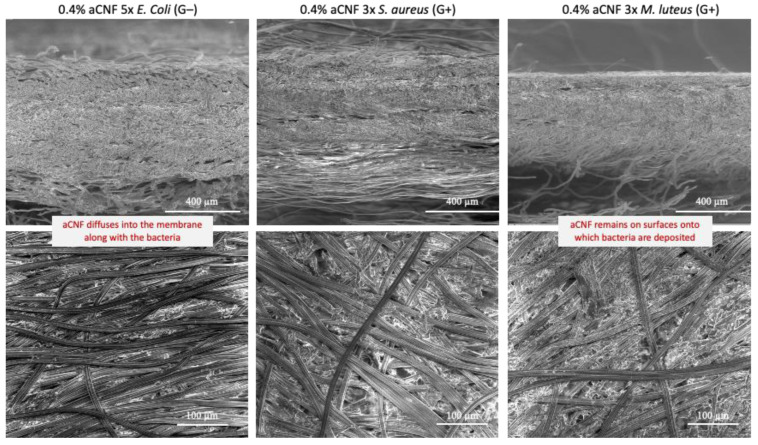
SEM micrographs for cross section (**top**) and surface (**bottom**) of an individual (single-layered) fibrous membrane (impregnated with 0.4 wt% of amino-hydrophobised CNF/aCNF, pre-suspended in 0.1 wt% quaternised cellulose nanofibrils/qCNF) after multi-filtration cycles of bacterial suspensions, performed with vacuum filtration at a pressure of 0.6 bar and room temperature.

The bacterial retention performance was also influenced by the concentration and distribution of aCNF in the fibrous membrane, which affected the thickness, areal and bulk densities of the membranes, providing more favourable conditions for the trapping of bacteria. However, depending on the type of membrane used (i.e., volumetric density and aCNF content), it was also evident that relatively hydrodynamically smaller bacterial cells (*S*. *aureus* and *E*. *coli*) diffuse into smaller pores and/or adsorb ionically/hydrophobically onto aCNF surfaces or become entrapped within, whereas larger bacterial cell clusters (with larger hydrodynamic volume, *M. luteus*) were eluted in the larger interfibrillar/fibre voids of the membrane (if they had not already been rejected on the membrane surface), which reduced the flow rates significantly. On the other hand, bacterial cells could also segregate according to their size as they moved through the membrane and elute in decreasing order of their hydrodynamic biovolume. Such an interpretation can also be supported by the SEM images presented in Figure 7, which show the extent of bacterial cell accumulation on the membrane surfaces in the case of larger clusters of *M. luteus* (also supported by photographs of individual membranes in Figure S3A), compared with the morphologically smaller clusters of *S. aureus* and *E. coli*, which can also get entrapped across the entire membrane structure (also supported by the photographs of the 4-layer structured membranes presented in Figures S3B and S4).

The results are in good agreement with other similar studies performing using CNF. A nano-to-microscale bilayer polymeric fibrous membrane with high charge density and a large volumetric surface area, containing TEMPO-oxidised CNF, was able to remove *E. coli* completely (log 4 reduction) by size-exclusion at a permeation rate of 89 L/h∗bar∗m^2^ [23]. A cationic-CNF shaped into 98% porous foam was able to achieve 85% antibacterial activity against *E. coli* and demonstrated good potential for air and liquid filtration with excellent absorbency through functional coating [24]. An electrospun CNF-PAN, containing silver nanoparticles, resulted in an excellent (91–99% reduction) bactericidal activity against *E. coli, Salmonella typhi* and *S. aureus* [13].

### 3.5. Retention Efficacy of E. coli Using Multi-Layer Sandwich-Structured Membranes with Different aCNF Content

**Figure 8:**
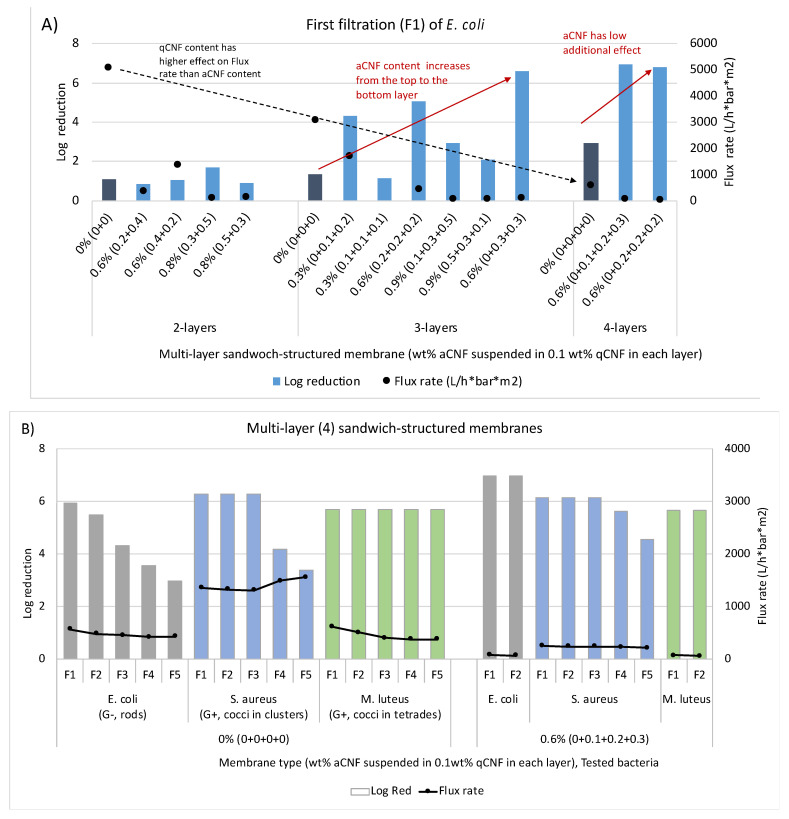
The effect of differently prepared (impregnated with different wt% of amino-hydrophobised CNF/aCNF, pre-suspended in 0.1 wt% quaternised cellulose nanofibrils/qCNF) sandwich (multi-layered) membrane on flux rate of (300 mL) bacterial suspension with log reduction values for (**A**) rod-shaped (G−) *Escherichia coli* (EXB-V127) vs. (**B**) round-shaped (G+) *Staphylococcus aureus* (EXB-V54) and *Micrococcus luteus* (EXB-V52), performed with vacuum filtration at a pressure of 0.6 bar and room temperature.

The results presented in Figure 8A show that the 3- and 4-layer structured membranes (log reduction 1.1–6) with increasing qCNF and aCNF contents induce a significantly better reduction of *E. coli* bacteria than 2-layer membranes (log reduction 0.8–1), which, however, slowed down the filtration process greatly (i.e., from about 5500 to 562 L/h∗bar∗m^2^ for membranes containing only qCNF in each layer). It is also evident that the combination of individual membranes, their impregnation with different aCNF content and their position in such a sandwich structure (i.e., from the top to the bottom) were equally important in increasing bacterial reduction, but without further significant reduction in the flow rate (i.e., up to 429–60 L/h∗bar∗m^2^). Moreover, the flow rate of bacterial suspension was not an indicator of the efficiency of bacterial removal from the aqueous suspension, but the position of the individual membranes containing aCNF was. Thus, a gradient-structured 3- or 4-layer membrane with equal content of aCNF (0.3 or 0.2 wt%, respectively) in the two or three layers from the bottom of the stack leads to excellent bacterial retention (up to 99.9999%, >6.6 log) at a >40 L/h∗bar∗m^2^ permeation rate. It is obvious that the filtration performance was improved by increasing the filtration depth and creating 3D structures. Single-layer membranes had fewer uniformly distributed pores/open spaces between the fibres and fibrils of qCNF/aCNF, which prevented the bacterial cells from being intercepted. In contrast, in the multi-layer structured membranes, a structural anisotropy with more open structure and lack of larger pore channels (as demonstrated by SEM imaging, Figure 3), due to different addition of aCNF, led to an improvement of homogeneity and, in combination with charged (CNF-rich) segments, to the improvement of the filtration performance (bacterial removal). 

Extremely high and composition-independent flux rates of bacterial suspensions (562–421 L/h∗bar∗m^2^ for *E. coli*, 1354–1550 L/h∗bar∗m^2^ for *S. aureus*, and 614–371 L/h∗bar∗m^2^ for *M. luteus*) were, therefore, measured for 4-layer structured membranes (Figure 8B), where each layer was impregnated with 0.1 wt% qCNF (in total 4 × 50 = 200 mg). This was the case even after the fifth filtration cycle (F1–F5), showing a high—although type-dependent—bacterial removal capacity (log reduction 5.9–2.9 for relatively hydrodynamically smaller rod-shaped *E. coli*, log reduction 6.2–3.3 for grape-like clusters of *S. aureus*, and log reduction 5.6–5.6 for hydrodynamically larger tetrads (or irregular clusters) of *M. luteus*), thus following the trend of rejection and capture properties being dependent on the surfaces and biovolumes of the bacterial cell clusters and their ability to undergo physiological change and orientation in the direction of flow, as discussed in the previous sections. Such a bacteria-dependent filtration effect was no longer evident when a 4-layer structured membrane was used that also contained (in addition to the qCNF) a gradient-increasing content of aCNF (from 0 to 0.3 wt%, totalling 0.6 wt% or 300 mg), resulting in an overall >5.6 log reduction of bacteria after two consecutive filtration cycles, albeit at slower flow rates (54–220 L/h∗bar∗m^2^). 

Although multi-layer filters are well known in the industry, very few studies have quantified their adsorption and filtration mechanisms systematically, and even fewer have characterised the effects of multi-layer structure on depth filter performance [55]. Bacterial filtration with multi-layer filters has been reported, with higher bacterial rejection achieved by attaching one layer to another mechanically, this latter also containing a bacteria-destroying material [56]. A nanofibre layer has also been attached to the first layer of a fibrous filter [57]. Multi-layer structured filters, where each layer has pore sizes/openings stacked on top of each other, also provide an easy way to separate cells sequentially [58], allowing the rapid optimisation of the number of filter layers based on the application needs.

## 4. Conclusions

The microfiltration membranes were prepared from differently oriented viscose fibre’ slivers, impregnated with quaternised (qCNF) and/or amino-hydrophobised (aCNF) cellulose nanofibrils, and, finally, compacted into fibrous bed membranes by vacuum filtration and cold pressing. The thickness, areal and gradient density, as well as structural morphology of the membranes varied due to the different proportions of the layers and addition of aCNF, as well as their further assembly into multi-layered sandwich structures. Filtration principles based on depth and surface filtration, size- and ionic/hydrophobic-adsorptions and the efficiency of each membrane layer, were confirmed by the filtration of spherical polystyrene particles of various micro sizes and surface chemistries. They also exhibited high capturing capacity for structurally and hydrodynamically different bacterial cells (*E. coli*, *S. aureus* and *M. luteus*) with simultaneous bacteriostatic (qCNF) and/or even bactericidal (aCNF) activity. The aCNF content reduced the water permeability and bacterial suspension flux rates, with lesser effects and greater stability in the case of multi-layered and aCNF content gradient-structured membrane, resulting in much higher (>5.6) log reductions in bacteria, even after the second–fourth filtration cycles, at relatively high and stable filtration rates (>50 L/h∗bar∗m^2^), thereby matching the performance of industrial polymeric filters for the removal of bacteria. The results demonstrated the potential usefulness of a simple and efficient approach for the rapid removal of bacteria from water, and possibly also from more the complex media used in downstream processes.

## Figures and Tables

**Table 1:** The list of viscous (VIS) fibre membranes used in the study and tested either as an individual (mono-layer) or sandwich (multi-layer) membranes. Each fibrous layer of approx. 0.43 g (or individual VIS slivers in the case of the 1-layer mixture/MIX) was impregnated with (in total) 50 mL of 0.1 wt% (i.e., 50 mg) quaternised cellulose nanofibrils (qCNF) without (0%), or with, the addition of 0.1–0.5 wt% (i.e., 50–250 mg) of amino-hydrophobised cellulose nanofibrils (aCNF).

(Total) Content (wt%,) of aCNF in Each Membrane Layer (or a VIS Sliver in a MIX), Stacked from Top to Bottom				
1-Layer/ Individual	2-Layer	3-Layer	4-Layer	1-Layer MIX
0	0 + 0	0 + 0 + 0	0 + 0 + 0 + 0	/
0.1	(0.3) 0.1 + 0.2	(0.3) 0 + 0.1 + 0.2	(0.6) 0 + 0.1 + 0.2 + 0.3	(0.6) 0 + 0.1 + 0.2 + 0.3
0.2	(0.6) 0.3 + 0.3	(0.3) 0.1 + 0.1 + 0.1	(0.6) 0 + 0.2 + 0.2 + 0.2	(0.6) 0.3 + 0.2 + 0.1 + 0
0.3	(0.6) 0.2 + 0.4	(0.6) 0 + 0.3 + 0.3		
0.4	(0.6) 0.4 + 0.2	(0.6) 0.2 + 0.2 + 0.2		
0.5	(0.8) 0.3 + 0.5	(0.6) 0.1 + 0.2 + 0.3		
	(0.8) 0.5 + 0.3	(0.6) 0.2 + 0.2 + 0.2		
		(0.9) 0.1 + 0.3 + 0.5		
		(0.9) 0.5 + 0.3 + 0.1		
		(0.9) 0.4 + 0.3 + 0.2		

## Data Availability

Not applicable.

## Supplementary Materials

The following supporting information can be downloaded at: https://www.mdpi.com/article/10.3390/membranes13030284/s1, Figure S1: SYTO9 and propidium iodide (PI) fluorescence viability staining with the LIVE/DEAD BacLight Bacterial Viability Kit (L7012, Invitrogen, Molecular probes) of selected individual fibrous membranes, namely, non-impregnated membrane (control) and membranes impregnated with 50 mL of 0.1% quaternised cellulose nanofibrils (qCNF) with or without the addition of 0.3% amino-hydrophobised CNF (aCNF); Figure S2: Morphology of Gram-stained stationary-phase bacterial cells of test strains used in the present study: (A) Escherichia coli (EXB-V127), (B) Staphylococcus aureus (EXB-V54), and (C) Micrococcus luteus (EXB-V52); Figure S3: Four individual (i.e., single-layer) fibrous membranes of the same type (each impregnated with 50 mL of 0.1 wt% quaternised cellulose nanofibrils) tested as 4-layer sandwich-structured membranes in a vacuum filtration performance assay with bacterial cell suspensions of (A) Micrococcus luteus (EXB-V52) and (B) Escherichia coli (EXB-V127); Figure S4: Four individual (single-layer) fibrous membranes (each impregnated with 50 mL of 0.1 wt% quaternised cellulose nanofibrils (qCNF), with and without the addition of 0.1, 0.2 or 0.3 wt% amino-hydrophobised CNF (aCNF)) after testing as 4-layer sandwich-structured membranes in a vacuum filtration performance assay with bacterial cell suspension of (A) Escherichia coli (EXB-V127) and (B) Staphylococcus aureus (EXB-V54); Table S1: Overview of the final nominal concentrations of the quaternised (qCNF) and amino-hydrophobised cellulose nanofibrils (aCNF) suspensions used in the broth macrodilution assay with the Gram-negative (G-) bacterial strain Escherichia coli (EXB-V127) and the Gram-positive (G+) bacterial strain Staphylococcus aureus (EXB-V54).
