# Supplementary material for: Cationised Fibre-Based Cellulose Multi-Layer Membranes for Sterile and High-Flow Bacteria Retention and Inactivation
Source: Membranes (Basel). 2023 Feb 27;13(3):284. doi: 10.3390/membranes13030284 (PMC10059598; doi:10.3390/membranes13030284)
Supplement: Supplementary file 1 [file membranes-13-00284-s001.zip › membranes-2218686-supplementary.pdf]

ELECTRONIC SUPPLEMENTARY MATERIAL

FOR:

# Cationised fibre-based cellulose multi-layer membranes for sterile and high-flow bacteria retention and inactivation

Vanja Kokol[1*], Monika Kos[2], Vera Vivod[1], Nina Gunde-Cimerman[2]

[1]University of Maribor, Faculty of Mechanical Engineering, Institute of Engineering Materials and Design, Smetanova ulica 17, 2000 Maribor, Slovenia, (vanja.kokol@um.si; vera.vivod@um.si)

[2]University of Ljubljana, Biotechnical Faculty, Department of Biology, Jamnikarjeva ulica 101, 1000 Ljubljana, Slovenia, (monika.kos@bf.uni-lj.si; nina.gunde-cimerman@bf.uni-lj.si)

The Electronic supplementary material comprises:

## 1. ASSESSMENT OF ANTIBACTERIAL PROPERTIES

### *1.1 Antimicrobial properties of qCNF and aCNF suspensions*

**Supplementary Table S1:** Overview of the final nominal concentrations of the quaternised (qCNF) and amino-hydrophobised cellulose nanofibrils (aCNF) suspensions used in the broth macrodilution assay with the Gram-negative (G-) bacterial strain *Escherichia coli* (EXB-V127) and the Gram-positive (G+) bacterial strain *Staphylococcus aureus* (EXB-V54).

### *1.2 Antimicrobial properties of the fibrous membranes: LIVE/DEAD viability assay*

**Supplementary Figure S1:** SYTO9 and propidium iodide (PI) fluorescence viability staining with the LIVE/DEAD BacLight Bacterial Viability Kit (L7012, Invitrogen, Molecular probes) of selected individual fibrous membranes, namely, non-impregnated membrane (control) and membranes impregnated with 50 mL of 0.1% quaternised cellulose nanofibrils (qCNF) with or without the addition of 0.3% amino-hydrophobised CNF (aCNF).

## 2. TEST MICROORGANISMS

**Supplementary Figure S2:** Morphology of Gram-stained stationary-phase bacterial cells of test strains used in the present study: (A) *Escherichia coli* (EXB-V127), (B) *Staphylococcus aureus* (EXB-V54), and (C) *Micrococcus luteus* (EXB-V52).

## 3. MEMBRANES FILTRATION EFFICACY USING BACTERIA CELLS

**Supplementary Figure S3:** Four individual (i.e. single-layer) fibrous membranes of the same type (each impregnated with 50 mL of 0.1 wt% quaternised cellulose nanofibrils) tested as 4-layer sandwich-structured membranes in a vacuum filtration performance assay with bacterial cell suspensions of (A) *Micrococcus luteus* (EXB-V52) and (B) *Escherichia coli* (EXB-V127).

**Supplementary Figure S4:** Four individual (single-layer) fibrous membranes (each impregnated with 50 mL of 0.1 wt% quaternised cellulose nanofibrils (qCNF), with and without the addition of 0.1, 0.2 or 0.3 wt% amino-hydrophobised CNF (aCNF)) after testing as 4-layer sandwich-structured membranes in a vacuum filtration performance assay with bacterial cell suspension of (A) *Escherichia coli* (EXB-V127) and (B) *Staphylococcus aureus* (EXB-V54).

# 1. ASSESSMENT OF ANTIBACTERIAL PROPERTIES

## *1.1 Antimicrobial properties of qCNF and aCNF suspensions*

**Supplementary Table S1:** Overview of the final nominal concentrations of the quaternised (qCNF) and amino-hydrophobised cellulose nanofibrils` (aCNF) suspensions used in the broth macrodilution assay with the Gram-negative (G-) bacterial strain *Escherichia coli* (EXB-V127) and the Gram-positive (G+) bacterial strain *Staphylococcus aureus* (EXB-V54).

| | **Final nominal concentration of the suspension in the test tube** (left column: mg/mL, right column: wt%) | | | |
|---|---|---|---|---|
| **Test sample** | **qCNF** | | **aCNF** | |
| (conc. of stock suspension)<br>**Dilution factor** | 12 mg/mL | 1.2 wt% | 32 mg/mL | 3.2 wt% |
| **Control** | 0 | 0 | 0 | 0 |
| **2-fold** | 6 | 0.6 | 16 | 1.6 |
| **4-fold** | 3 | 0.3 | 8 | 0.8 |
| **8-fold** | 1.5 | 0.15 | 4 | 0.4 |
| **16-fold** | 0.75 | 0.075 | 2 | 0.2 |
| **32-fold** | - | - | 1 | 0.1 |
| **64-fold** | - | - | 0.5 | 0.05 |

*1.2 Antimicrobial properties of the fibrous membranes: LIVE/DEAD viability assay*

**Supplementary Figure S1:** SYTO9 and propidium iodide (PI) fluorescence viability staining with the LIVE/DEAD BacLight Bacterial Viability Kit (L7012, Invitrogen, Molecular probes) of selected fibre membranes, namely, a non-impregnated membrane (control) and membranes impregnated with 50 mL of 0.1% quaternised cellulose nanofibrils (qCNF) with or without the addition of 0.3% amino-hydrophobised CNF (aCNF).

Staining was applied immediately after vacuum filtration of 200 mL of an *Escherichia coli* (EXB V127) aqueous suspension (concentration of bacteria: ~$10^6$ CFU/mL) through individual fibrous membranes at a vacuum pressure of 0.6 bar and room temperature. The fluorescence was measured spectroscopically with a Biotek Cytation Hybrid Multimode Reader (Agilent, CA, USA) by using an excitation wavelength centred at 475 nm, while the relative fluorescence intensities were acquired of the green (Syto9 emission at 505 nm) and red (PI emission at 617 nm) emissions. The stacked bar graph shows the average Relative Fluorescence Units (RFU) of the green and red emissions of the unstained and stained membranes. The graph also includes the calculated values after subtracting the signal of unstained membranes from the stained membranes.

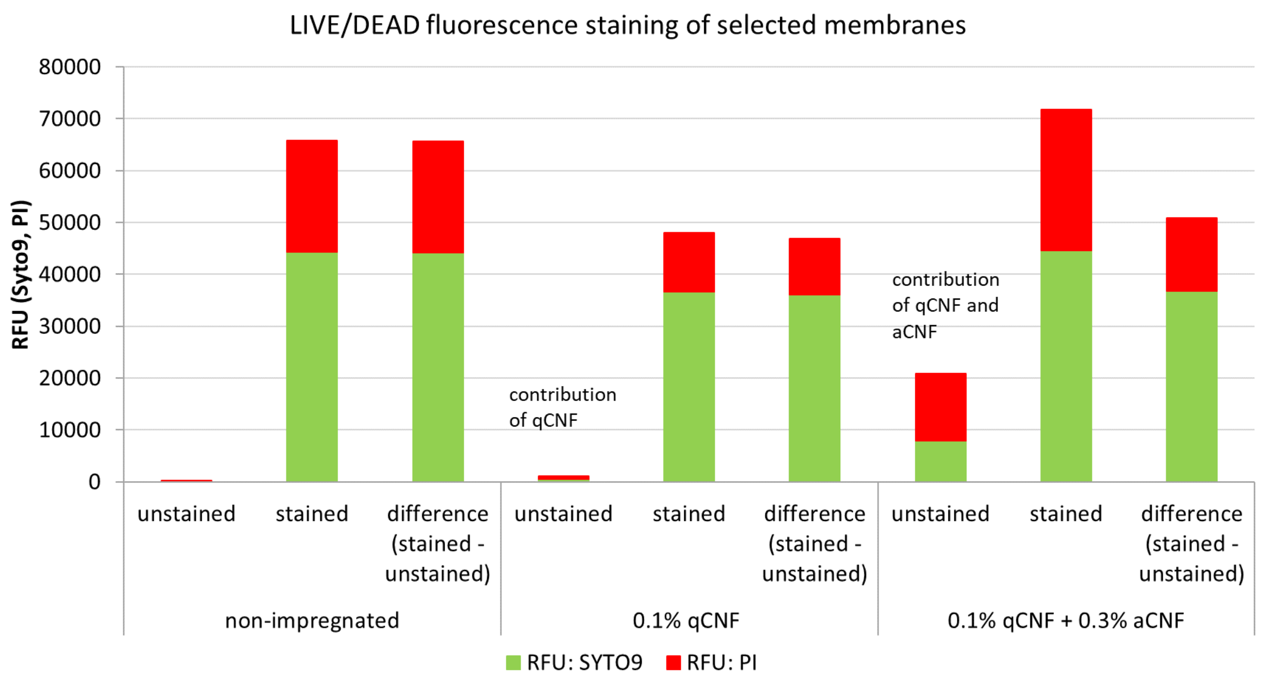

## 2. TEST MICROORGANISMS

**Supplementary Figure S2:** Morphology of the Gram stained stationary-phase bacterial cells of test strains used in the present study: (A) *Escherichia coli* (EXB-V127), (B) *Staphylococcus aureus* (EXB-V54), and (C) *Micrococcus luteus* (EXB-V52).

The strains were grown in tryptic-soy-broth and incubated overnight (for 16 h *E. coli* and *S. aureus*; and for 18 h *M. luteus*) at 37±0.5 °C under dynamic conditions. For the Gram staining, the bacterial cell suspension was prepared in deionised water and the cell concentration was adjusted to ~$10^8$ CFU/mL. The images were acquired with an Olympus BX51 light microscope at 1000x magnification using the oil immersion technique. The Gram-negative (G-) bacteria *E. coli* were stained palish red, while the Gram-positive (G+) bacteria *S. aureus* and *M. luteus* were stained purple. The size, shape and arrangement of the cells differed significantly: The *E. coli* is characterised by single rod-shaped cells (bacilli), whereas the cells of the other two bacteria are spherical (cocci), and often arranged in groups of different sizes (pairs – diplococci, tetrads, or irregular grape-like clusters).

A)

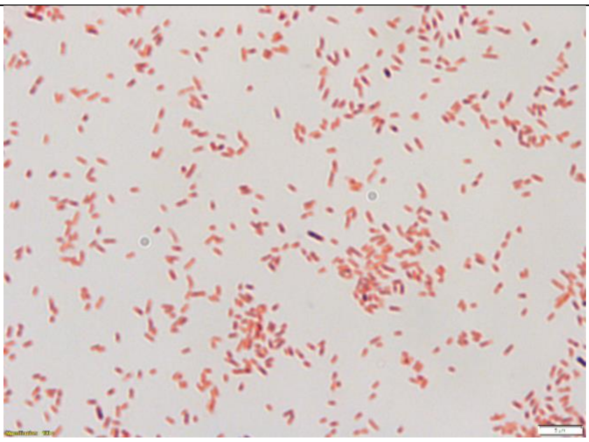

**Gram-negative rods of *Escherichia coli* (EXB-V127).**

Oil immersion light microscopy
Magnification: 1000x
Scale bar: 5 µm

B)

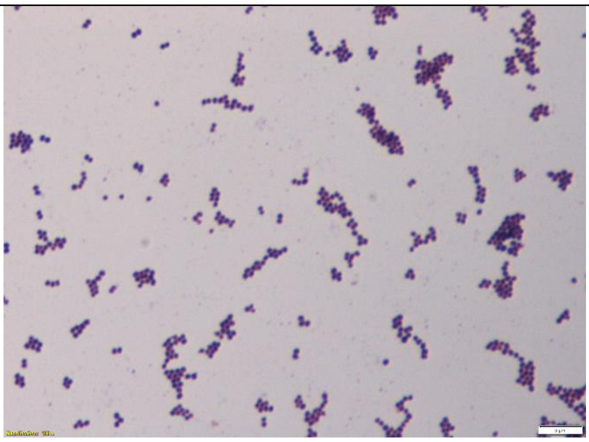

**Gram-positive cocci of *Staphylococcus aureus* (EXB-V54), which form mostly irregular grape-like clusters**

Oil immersion light microscopy
Magnification: 1000x
Scale bar: 5 µm

| | | |
|---|---|---|
| C) | 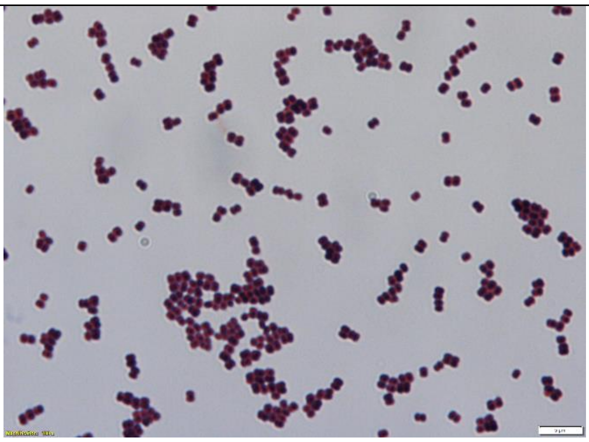 | **Gram positive cocci, arranged mostly in tetrads, of *Micrococcus luteus* (EXB-V52).**<br><br>Oil immersion light microscopy<br>Magnification: 1000x<br>Scale bar: 5 µm |

## 3. MEMBRANES` FILTRATION EFFICACY USING BACTERIA CELLS

**Supplementary Figure S3:** Four individual (i.e. single-layer) fibrous membranes of the same type (each impregnated with 50 mL of 0.1 wt% quaternised cellulose nanofibrils) tested as 4-layer sandwich-structured membranes in a vacuum filtration performance assay with bacterial cell suspension of (A) *Micrococcus luteus* (EXB-V52) and (B) *Escherichia coli* (EXBV127).

For each sandwich membrane, five consecutive filtrations of 200 mL of the feed suspension (concentration of the bacteria ~ $10^6$ CFU/mL) were performed at a vacuum pressure of 0.6 bar and at room temperature.

**A)** After completion of the filtration of *M. luteus* feed suspension, a yellow filter cake was observed on the outside of the upper surface membrane ($1^{st}$/4, red circle). The yellow colour originated from the concentrated stationary-phase *M. luteus* cells (A-i). Such visual observation was not confirmed on the other three underlying membranes ($2^{nd}$-$4^{th}$/4).

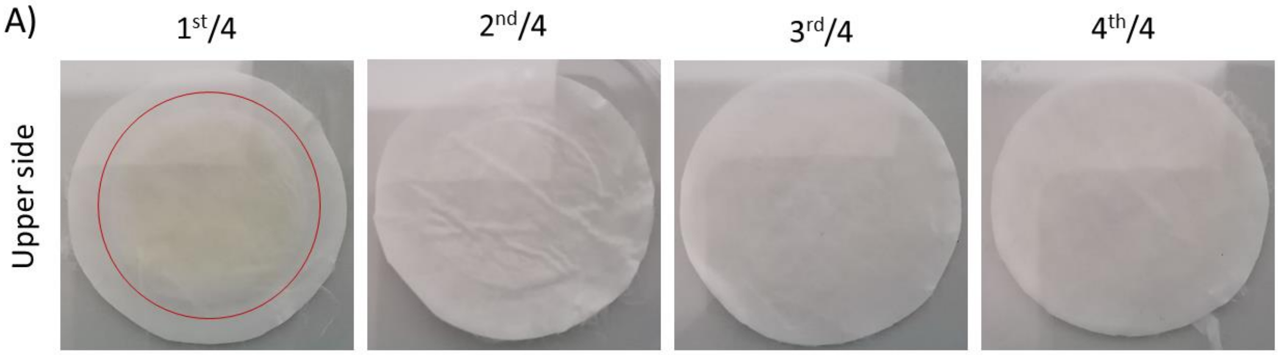


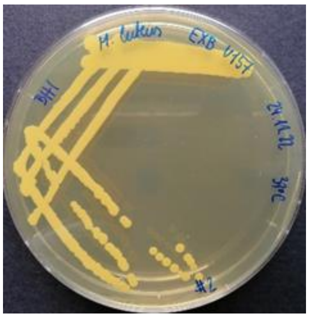

**A-i)**

*M. luteus* (EXB-V52) grown on a BHI medium forms circular, entire, convex, and creamy yellow-pigmented colonies with diameters of approximately 3-4 mm after 2 days at 37 °C.

**B)** However, in the case of *E. coli*, whose concentrated cells appeared greyish-white (B-i), the filter cake could not be distinguished from the white base of the upper membrane ($1^{st}$/4) or underlying membranes ($2^{nd}$–$4^{th}$/4). The brown stains on the edge of the upper membrane $1^{st}$/4) are the result of contact with the hot tweezers at the end of the filtration experiment.

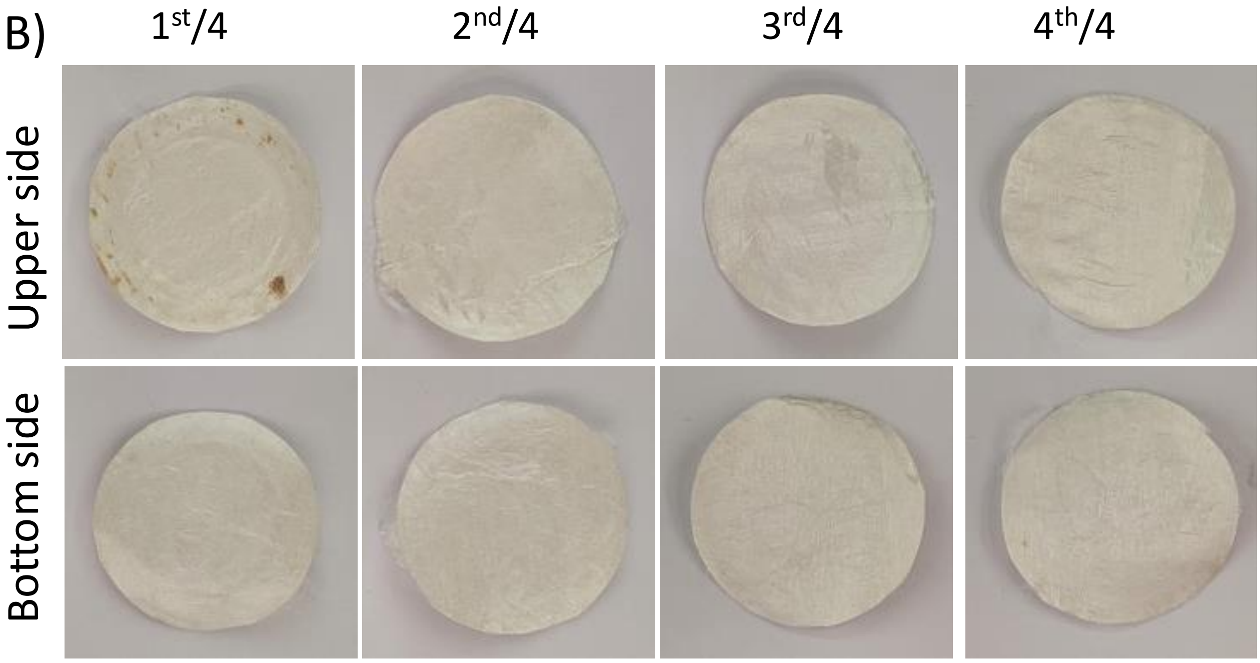


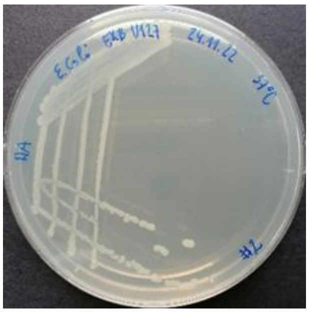

**B-i)**

*E. coli* (EXB-V127) grown on nutrient agar forms circular, entire, moist, smooth, opaque, greyish-white colonies with diameters of a few mm after 1 day at 37 °C.

**Supplementary Figure S4:** Four individual (single-layer) fibrous membranes (each impregnated with 50 mL of 0.1 wt% quaternised cellulose nanofibrils (qCNF), with and without the addition of 0.1, 0.2 or 0.3 wt% amino-hydrophobised (aCNF)) after testing as 4-layer sandwich-structured membranes in a vacuum filtration performance assay with bacterial cell suspension of (A) *Escherichia coli* (EXB-V127) and (B) *Staphylococcus aureus* (EXB-V54).

For each sandwich membrane, two (*E. coli*) or five (*S. aureus*) consecutive filtrations of 200 mL of the feed suspension (concentration of the bacteria ~ $10^6$ CFU/mL) were performed at a vacuum pressure of 0.6 bar and at room temperature.

**A and B)** After completion of the experiment, no apparent filter cakes could be distinguished on the upper side of the first membranes or underlying membranes, since concentrated cells of both strains appeared in shades of white. The brownish stains on individual membranes were attributed to the higher concentration of aCNF.

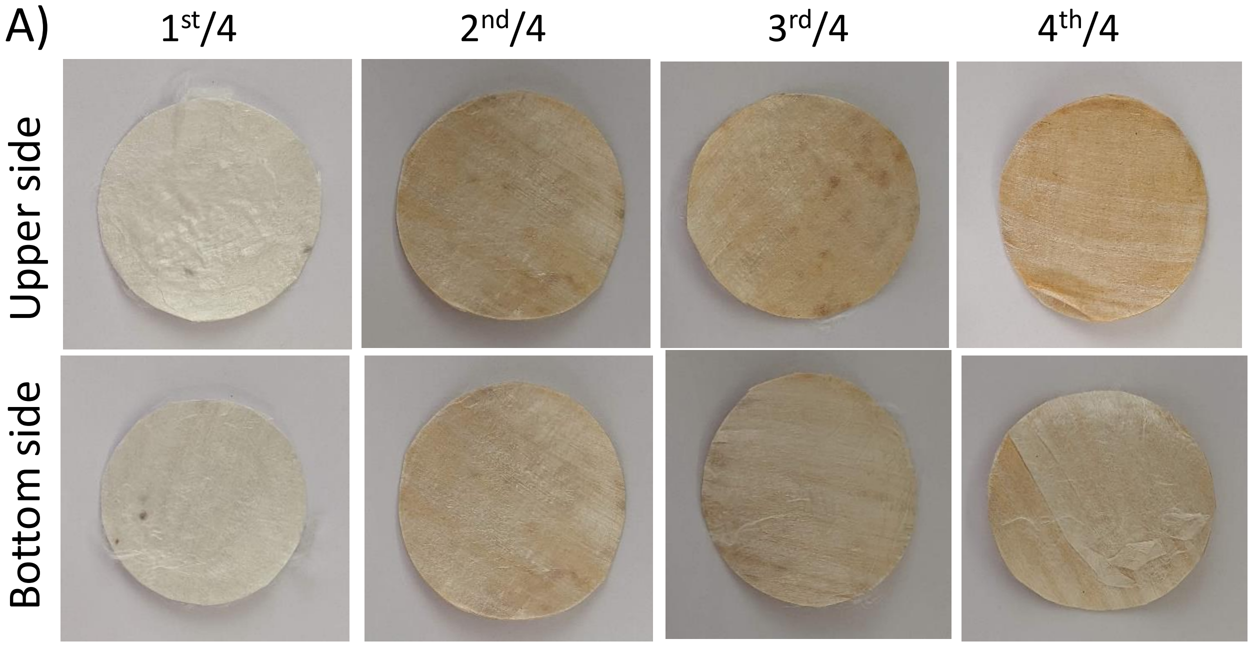

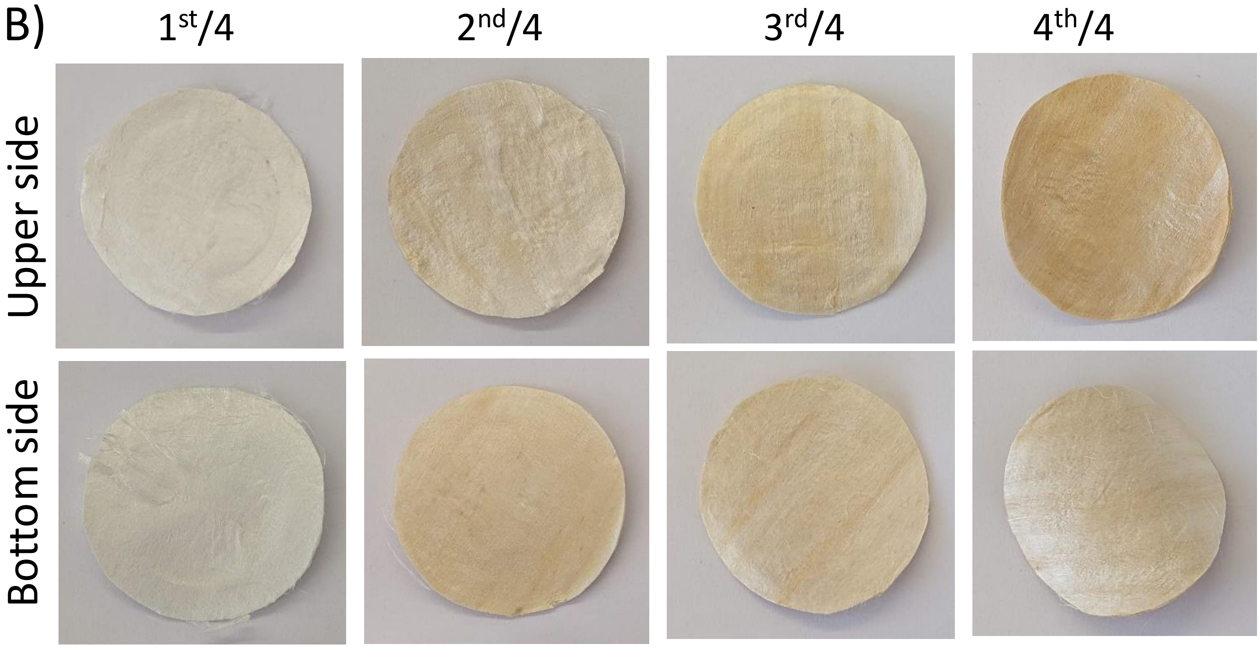


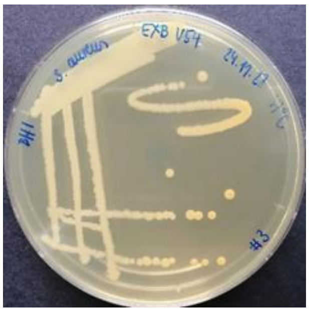


**B-i)**

*S. aureus* (EXB-V54) grown on BHI agar form circular, entire, raised, smooth, opaque, creamy-white colonies with diameters of 2-3 mm after 2 days at 37 °C.
